# Heterologous Expression Reveals Ancient Properties of Tei3—A VanS Ortholog from the Teicoplanin Producer *Actinoplanes teichomyceticus*

**DOI:** 10.3390/ijms232415713

**Published:** 2022-12-11

**Authors:** Oleksandr Yushchuk, Kseniia Zhukrovska, Bohdan Ostash, Victor Fedorenko, Flavia Marinelli

**Affiliations:** 1 Department of Biotechnology and Life Sciences, University of Insubria, 21100 Varese, Italy; 2 Department of Genetics and Biotechnology, Ivan Franko National University of Lviv, 79005 Lviv, Ukraine

**Keywords:** *Actinoplanes teichomyceticus*, VanS, glycopeptide antibiotic resistance, teicoplanin, A40926

## Abstract

Glycopeptide antibiotics (GPAs) are among the most clinically successful antimicrobials. GPAs inhibit cell-wall biosynthesis in Gram-positive bacteria via binding to lipid II. Natural GPAs are produced by various actinobacteria. Being themselves Gram-positives, the GPA producers evolved sophisticated mechanisms of self-resistance to avoid suicide during antibiotic production. These self-resistance genes are considered the primary source of GPA resistance genes actually spreading among pathogenic enterococci and staphylococci. The GPA-resistance mechanism in *Actinoplanes teichomyceticus*—the producer of the last-resort-drug teicoplanin—has been intensively studied in recent years, posing relevant questions about the role of Tei3 sensor histidine kinase. In the current work, the molecular properties of Tei3 were investigated. The setup of a GPA-responsive assay system in the model *Streptomyces coelicolor* allowed us to demonstrate that Tei3 functions as a non-inducible kinase, conferring high levels of GPA resistance in *A. teichomyceticus*. The expression of different truncated versions of *tei3* in *S. coelicolor* indicated that both the transmembrane helices of Tei3 are crucial for proper functioning. Finally, a hybrid gene was constructed, coding for a chimera protein combining the Tei3 sensor domain with the kinase domain of VanS, with the latter being the inducible Tei3 ortholog from *S. coelicolor.* Surprisingly, such a chimera did not respond to teicoplanin, but indeed to the related GPA A40926. Coupling these experimental results with a further in silico analysis, a novel scenario on GPA-resistance and biosynthetic genes co-evolution in *A. teichomyceticus* was hereby proposed.

## 1. Introduction

Glycopeptide antibiotics (GPAs) [1], particularly dalbaheptides [2], belong to a clinically relevant group of natural compounds produced by soil-dwelling and mycelium-forming Gram-positive actinobacteria, traditionally named actinomycetes [3,4]. As with other natural compounds, genes responsible for the biosynthesis of GPAs are grouped in large biosynthetic gene clusters (BGCs) [5]. GPAs are successfully utilized to treat severe infections caused by multi-drug-resistant (MDR) staphylococcal and enterococcal strains. Two natural and three semisynthetic GPAs are currently approved for clinical use [3,6,7]. Natural first-generation GPAs are vancomycin and teicoplanin, produced by the actinobacteria *Amycolatopsis orientalis* (various strains) [8] and *Actinoplanes teichomyceticus* ATCC 31121 [9], respectively. Vancomycin was introduced in clinics first (in 1958), followed by teicoplanin (1988) in Europe and then in Japan (1998) [3]. Semisynthetic second-generation GPAs include telavancin—a vancomycin derivative [10], oritavancin—a derivative of the natural GPA chloroeremomycin [11], and dalbavancin—a derivative of the other natural GPA, A40926 [12].

Although GPAs of both generations are still clinically useful, resistant pathogens are inevitably emerging [13,14,15,16]. Peculiarly, resistance mechanisms in GPA producers and pathogens are very similar [17]. It is likely that pathogens acquired GPA resistance genes from GPA producers, or more generally from non-producing actinobacteria, where GPA resistance determinants are abundant [18,19]. GPAs interact with d-alanyl-d-alanine (d-Ala-d-Ala) termini of the nascent peptidoglycan (PG), impeding upstream transpeptidation and transglycosylation reactions and consequently blocking cell wall biosynthesis [20]. The resistance mechanisms shared between GPA producers and pathogens lead to the modification of the d-Ala-d-Ala termini, reducing the affinity of GPAs for their target. d-Ala-d-Ala termini in PG precursors are replaced by d-Ala-d-lactate (d-Lac) [17,21] or truncated to a single d-Ala residue [22,23,24]. The first mechanism requires a three-gene operon—*vanHAX,* where *vanH* codes for a d-Lac dehydrogenase responsible for generating a d-Lac pool, *vanX*—for a d,d-dipeptidase whose role is reducing the intracellular pool of d-Ala-d-Ala [25,26], and *vanA*—for a d-Ala-d-Lac ligase [21,27]. The second involves a single d,d-carboxypeptidase named VanY, which trims the terminal d-Ala residue. In both cases, PG precursors still undergo transpeptidation reactions, but this process is not affected by GPAs [22,23,24]. The expression of *vanY* or *vanHAX* might be constitutive or inducible. The latter requires a two-component regulatory system consisting of a sensor histidine kinase (SHK) and a transcriptional response regulator (RR), also known as VanS and VanR, respectively [28,29,30,31]. VanS can specifically recognize extracellular GPAs and phosphorylate VanR, which, in turn, activates the expression of other *van* genes [29,30,31,32,33].

Actinobacterial GPA producers such as *A. teichomyceticus, Amycolatopsis balhimycina* DSM 5908 (balhimycin producer), *Nonomuraea gerenzanensis* ATCC 39727 (A40926 producer), *Streptomyces toyocaensis* NRRL 15009 (A47934 producer), as well as the GPA non-producing *Streptomyces coelicolor* A3(2) represent well-studied models of GPA resistance mechanisms (recently reviewed in [34]), although certain aspects are not fully elucidated yet and merit further investigation. In this work, the properties of Tei3—the VanS ortholog from the teicoplanin producer *A. teichomyceticus*—were investigated in vivo. Notably, *A. teichomyceticus* is resistant to very high concentrations of teicoplanin, likely due to the strong constitutive expression of the *vanHAX* orthologs—*tei7-6-5*, which was previously reported during the transcription analyses of the *tei* BGC [35,36,37]. It was speculated that Tei3 acts as a constitutive SHK [35]. We aimed to experimentally investigate whether Tei3 really acts as a constitutive SHK and if any GPA is eventually recognized by its sensor domain (SD). Conducting a series of experiments in *S. coelicolor* as a heterologous host, it was found out that Tei3 does not require any ligand to function. Strikingly, the SD of Tei3 appeared to recognize not teicoplanin but the structurally related GPA A40926. Finally, reconstruction of the phylogeny of glycosyltransferases (GTFs) coded within GPA BGCs allowed us to build a possible scenario explaining the obtained results.

## 2. Results

The main goals of this work were to test if Tei3 acts as a non-inducible phosphorylase and to investigate which ligand could be sensed by Tei3. Considering *A. teichomyceticus* a challenging microorganism for gene-engineering manipulations, a series of genetic experiments were designed and performed in heterologous hosts such as *S. coelicolor* M512 [38] and *S. coelicolor* J3200 [31]. As anticipated in the introduction, *S. coelicolor* has a full set of *van* genes conferring vancomycin resistance, although it does not produce any GPAs [39]. *S. coelicolor* M512 does not produce the pigmented antibiotics actinorhodin and undecylprodigiosin [38], permitting the use of a β-glucuronidase (GusA)-based reporter assay, as described below. The second strain—J3200—is the Δ*vanS_Sc_* mutant, in which the host *vanS* gene was knocked out [31]. Thus, the experimental steps described below aimed: (1) to test if the heterologous Tei3 can cross-phosphorylate the host VanR_Sc_; (2) to build a *gusA*-based reporter *S. coelicolor* strain, able to convert X-Gluc (5-bromo-4-chloro-3-indolyl-β-d-glucuronide) to 5,5′-dibromo-4,4′-dichloro-indigo following GPA induction; (3) to utilize the created reporter strain to show if Tei3 needs the presence of GPAs or not; (4) to replace the SD of the host VanS_Sc_ with its counterpart from Tei3 and test which GPAs may act as ligands for Tei3 SD.

### 2.1. Heterologous Expression of Tei3 SHK Leads to Teicoplanin and A40926 Resistance in S. coelicolor M512

**Figure 1:**
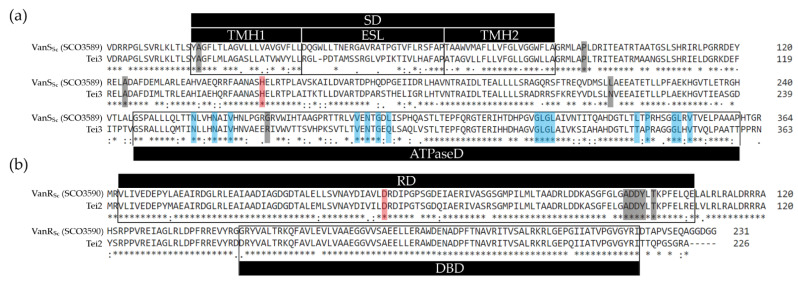
Clustal Omega [41] pairwise alignments of aa sequences of Tei3 and VanS_Sc_ (**a**) as well as of Tei2 and VanR_Sc_ (**b**). Domains and sequence features were annotated according to [31] and CD-Search tool [42]; SD—sensor domain, TMH1/2—first and second transmembrane α-helices, ESL—extracytoplasmic sensory loop, ATPaseD—ATPase domain, RD—response domain, DBD—DNA binding domain. In (**a**): mutation sites that were shown to impair VanS_Sc_ function [31] are highlighted in gray; the autophosphorylation site is highlighted in red; conserved ATP-binding sites are in blue. In (**b**): residues putatively involved in dimerization are highlighted in gray, while the phosphorylation site is in red.

VanRS-like two-component regulatory pairs are conserved enough among actinobacteria to show a certain degree of cross-talking [33,40]. For example, it was shown that VanR_Sc_ (coming from *S. coelicolor*) could be phosphorylated in vivo by VanS_St_ (from A47934 producer *S. toyocaensis*), but not vice versa, implying that VanR_Sc_ is accessible for non-cognate SHKs [33]. In this case, SHKs and RRs both came from *Streptomyces* spp. Instead, it was necessary to check if Tei3 SHK from *A. teichomyceticus* (order *Micromonosporales*) could phosphorylate VanR_Sc_. Overall, Tei2 and Tei3 share a high percentage of aa sequence identity with VanR_Sc_ and VanS_Sc_: 91% and 67%, respectively. Tei3 and VanS_Sc_ are collinear, sharing highly similar transmembrane helix (TMHs) regions and conserved putative autophosphorylation sites (Figure 1a). The most divergent region between Tei3 and VanS_Sc_ is the extracytoplasmic sensory loop (ESL), implying that quite different ligands should be recognized by the two proteins (Figure 1a). At the same time, Tei2 and VanR_Sc_ were almost identical (Figure 1b). Thus, it seems plausible that Tei3 would be able to phosphorylate VanR_Sc_ in vivo.

**Figure 2:**
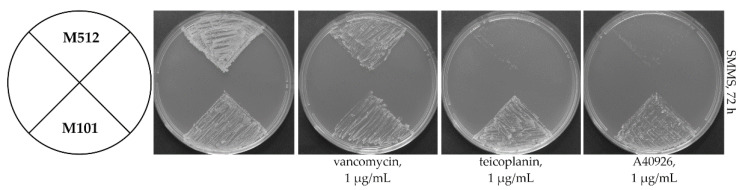
Expression of *tei3* led to teicoplanin and A40926 resistance in *S. coelicolor* M512. A number of 10^6^ spores of *S. coelicolor* M512 and M101 were inoculated at each sector of SMMS agar plates added with vancomycin, teicoplanin, and A40926. Plates were examined after 72 h of incubation. *S. coelicolor* M512 was resistant to vancomycin and sensitive to teicoplanin and A40926, as previously reported [43], whereas M101 was resistant to vancomycin, A40926, and teicoplanin.

To test if Tei3 can phosphorylate the non-cognate RR—VanR_Sc_, *tei3* was cloned into the pSET152A vector, giving pGP101, and then transferred into *S. coelicolor* M512. The obtained recombinant strain—*S. coelicolor* M101—gained teicoplanin and A40926 resistance, in addition to the endogenous vancomycin resistance (Figure 2). Consequently, it could be concluded that Tei3 phosphorylates VanR_Sc_ in vivo. However, this experiment did not clarify whether Tei3 acts as a constitutive phosphorylase, considering that teicoplanin and A40926 added to the plates could serve as its activators.

### 2.2. Tei3 Acts as a Non-Inducible Phosphorylase

Expression of *tei2-3-4* and *tei7-6-5* operons remains stable throughout the life cycle of *A. teichomyceticus,* independently from the teicoplanin concentration [35,37], pathway-specific regulation [36], and growth phase [37]. Previous papers speculated about the need of a strong constitutive promoter driving *tei2-3-4* expression [37] or of the presence of specific mutations in Tei3 that might explain its function as a constitutive phosphorylase, working independently from the presence of any extracellular GPA [35]. Protein sequence comparison indicated that Tei3 carries single aa substitutions in two sites where analogous mutations transform the vancomycin-inducible VanS_Sc_ into a constitutive phosphorylase [35]. Specifically, these are L216P and G271V substitutions [31]. Homologous sites in Tei3 are N215 and R270 (Figure 1a). However, there are multiple other sites within the putative ATPase domain (ATPaseD) of Tei3, which significantly diverged from VanS_Sc_ (see Figure 1a).

**Figure 3:**
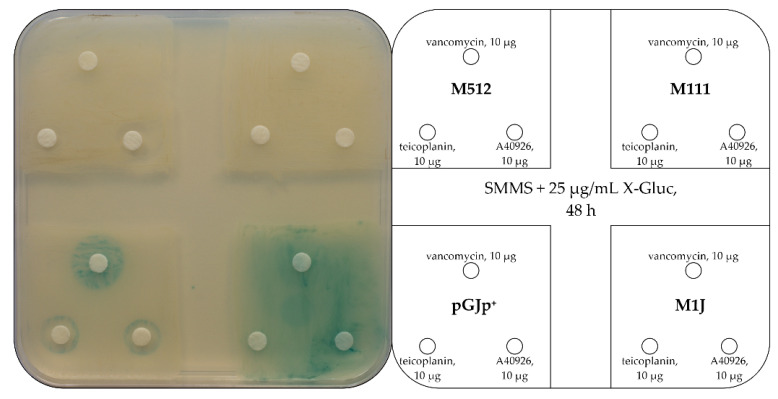
*gusA*-based GPA-inducible reporter assay revealed a constitutive phosphorylase activity of Tei3. *S. coelicolor* strains pGJp^+^ and M1J were inoculated on SMMS agar added with 25 µg/mL of X-Gluc along with their *gusA*-control strains, i.e., the teicoplanin/A40926-sensitive M512 and the teicoplanin/A40926-resistant M111. Notably, in pGJp^+^, the chromogenic conversion of X-Gluc (observed as green halos) occurred only upon GPA induction, while the background of M1J remained completely green, implying that Tei3 did not require GPA induction of its activity. A number of 10^7^ spores of each strain were used for inoculation and the plate was examined after 48 h of incubation.

To experimentally verify the GPA inducibility of Tei3, a bioassay responding to the inducers of *van* genes was first developed in *S. coelicolor*. Other authors previously described a reporter *S. coelicolor* strain, where the endogenous *vanJ* promoter (*vanJp*) was cloned into a multicopy plasmid, fused with the kanamycin/neomycin resistance gene—*neo*—in a way that induction of *vanJp* by vancomycin conferred resistance to both neomycin and kanamycin [39]. Using this experience, either *vanJp* or the other endogenous vancomycin-responsive *S. coelicolor vanHAX* promoter (*vanHp*) was fused with the *gusA* gene, coding for a β-glucuronidase in the pGUS chassis [44]. *vanJp* or *vanHp* activation by GPAs in the reporter strain should activate the chromogenic conversion of the X-Gluc substrate into the green-colored 5,5′-dibromo-4,4′-dichloro-indigo [44]. Thus, plasmids pGHp (carrying *vanHp-gusA*) and pGJp (carrying *vanJp-gusA*) were transferred to *S. coelicolor* M512 by means of intergeneric conjugation with *Escherichia coli* ET12567 pUZ8002^+^. The inducibility of the two generated reporter strains *S. coelicolor* pGHp^+^ and pGJp^+^ was first tested in liquid medium and then in solid plates. When vancomycin was added at 10 µg/mL to 50 h old cultures in TSB liquid medium, the basal glucuronidase activity of the mycelia was increased by, ca., twenty- and forty-fold, in *S. coelicolor* pGHp^+^ and pGJp^+^, respectively (Figure S1), indicating that *vanJp* seems more responsive to vancomycin than *vanHp.* In solid media containing 25 µg/mL of X-Gluc, the presence of vancomycin induced *vanJp*-mediated glucuronidase activity in recombinant strains, yielding green halos around the Whatman discs soaked in antibiotic solution (Figure 3 and Figure S2). *S. coelicolor* pGJp^+^ acted very well as a reporter, giving a detectable chromogenic conversion in response to very low concentrations of vancomycin (250 ng, Figure S2), whereas *S. coelicolor* pGHp^+^ resulted as much less responsive to vancomycin (data not shown) and, thus, it was not further used.

The next step was transferring Tei3 into *S. coelicolor* pGJp^+^ to test if its activity is inducible by GPAs. As either pGJp or pGP101 is a distant derivative of pSET152 [45], both plasmids use the φC31 *attB* site for integration and are not compatible. To solve this issue, the pRT801 plasmid [46] was used to create a φBT1-based derivative of pGP101, named pGP111. The M512 derivative carrying pGP111 (carrying *tei3*) was named M111, while the strain carrying both pGP111 and pGJp was named M1J. It was expected that if Tei3 functions as a constitutive phosphorylase, M1J would express a constitutive glucuronidase activity in the presence of X-Gluc, not depending on the presence or absence of GPAs. This assumption was correct as M1J converted X-Gluc independently from the presence of any GPA (Figure 3), confirming in vivo that Tei3 functions as a constitutive phosphorylase. As a control, it was shown that no induction was observed in those strains not carrying *vanJp-gusA* (M512 and M111), whereas the GPA induction was observed in *S. coelicolor* pGJp^+^ due to the action of the endogenous VanS_Sc_.

### 2.3. Loss of Extracytoplasmic Sensory Loop and Transmembrane Helices Renders Tei3 and VanS_Sc_ Nonfunctional

**Figure 4:**
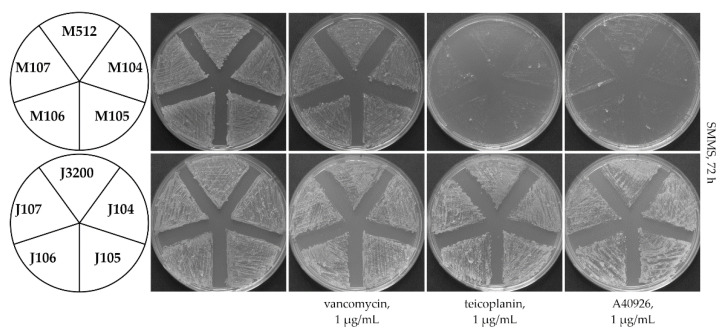
Loss of the gene fragments coding for transmembrane helices and extracytoplasmic sensory loops makes *tei3* and *vanS_Sc_* non-functional. A number of 10^6^ spores were inoculated at each sector of SMMS agar plates added with vancomycin, teicoplanin, or A40926; plates were examined after 72 h of incubation. All the recombinant strains deriving from M512 were vancomycin-resistant and teicoplanin- and A40926-sensitive, whereas all the recombinant strains deriving from J3200 were resistant to all the tested GPAs.

Considering the non-inducible properties of Tei3, we were wondering whether TMHs and ESL are still necessary for its function, or whether ATPaseD would be the only required domain. To answer this question, a series of plasmids was created carrying truncated versions of *tei3* or of *vanS_Sc_* lacking (i) TMH1 and ESL (*tei3*′, *vanS_Sc_*′); (ii) TMH1, ESL, and TMH2 (*tei3*″, *vanS_Sc_*″) (Figure S3). These plasmids were named pGP104 (*tei3*′), pGP105 (*vanS_Sc_*′), pGP106 (*tei3*″), and pGP107 (*vanS_Sc_*″). Plasmids were transferred into *S. coelicolor* M512 (resistant to vancomycin but sensitive to teicoplanin and A40926) and *S. coelicolor* J3200 (this last one—Δ*vanS_Sc_*—is constitutively resistant to all GPAs), generating M104-107 and J104-107 strains. It was expected that if ATPaseD of Tei3 would be able to function alone, the expression of *tei3*′ and *tei3*″ might lead to constitutive GPA resistance in M512. On the contrary, the absence of the SD in VanS_Sc_ might render J3200 constitutively sensitive to GPAs. However, the obtained results indicated that ATPaseDs of neither Tei3 nor VanS_Sc_ can function alone: the phenotypes of M104-107 and J104-107 strains in the presence of vancomycin, teicoplanin, and A40926 were the same as the parental M512 and J3200 strains, respectively (Figure 4).

### 2.4. Tei3 Sensor Domain Is Sensitive to A40926 but Not to Teicoplanin and Vancomycin

**Figure 5:**
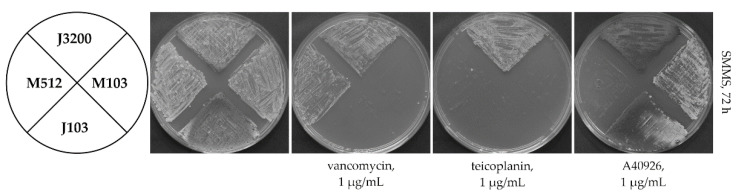
Hybrid SHK containing SD from Tei3 and ATPaseD from VanS_Sc_ made *S. coelicolor* J3200 sensitive to teicoplanin and vancomycin, and *S. coelicolor* M512 sensitive to vancomycin, but resistant to A40926, implying that Tei3 SD recognized A40926 as a ligand. A number of 10^6^ spores were inoculated at each sector of SMMS agar plates added with vancomycin, teicoplanin, or A40926; plates were examined after 72 h of incubation.

Logically, teicoplanin should be the original ligand for Tei3. However, direct experimental verification of this assumption is impossible due to the non-inducible properties of Tei3. It is possible that maybe Tei3 became a constitutive phosphorylase in the course of the evolution, but clues for Tei3 sensitivity might have remained “fossilized” within its SD. To answer this question, a hybrid gene coding for a chimeric SHK combining the SD of Tei3 with the ATPaseD of VanS_Sc_ was created. *vanS_Sc_* and *tei3* (Figure S4) were collinear, facilitating such an exchange. Luckily, a unique *Pae*I recognition site was found only 4 bp after the *vanS_Sc_* region coding for SD. This allowed us to use this site for replacing the *vanS_Sc_* region coding for the SD with the corresponding one from *tei3* (see Section 4 for details). The obtained hybrid gene—*tei3-vanS_Sc_* (Figure S4)—was cloned into the pSET152A plasmid generating pGP103. Next, pGP103 was transferred into M512 and J3200 generating the recombinant strains M103 and J103, respectively. In the first case, a merodiploid strain was generated, carrying the native *vanS_Sc_* together with the hybrid *tei3-vanS_Sc_* allele, while in J3200, the knockout of *vanS_Sc_* was complemented by the added *tei3-vanS_Sc_*. GPA resistance phenotypes of these recombinants showed that both M103 and J103 became sensitive to vancomycin, implying that this GPA is not an inducer for the Tei3 SD (Figure 5).

Surprisingly, M103 and J103 were also sensitive to teicoplanin, excluding its possible role as a ligand for the Tei3 SD. Adding a final detail to these puzzling results, both M103 and J103 were resistant to A40926, implying that the Tei3 SD recognizes A40926 as a ligand.

### 2.5. Establishing a Link between the Evolution of Glycosylation Pattern of Teicoplanin and Properties of Tei3 SD

**Figure 6:**
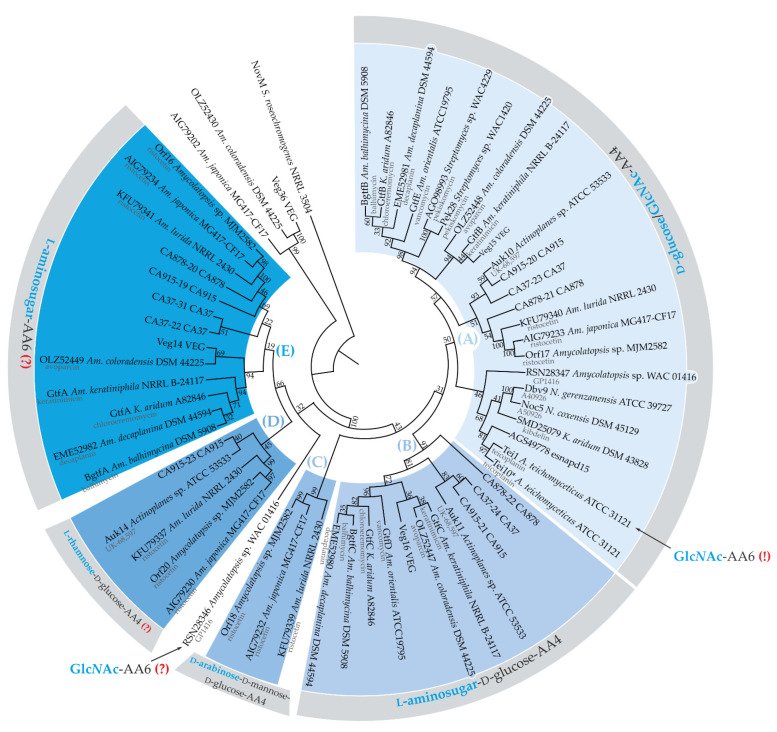
Maximum-likelihood phylogenetic tree of 59 GTFs coded within or near experimentally studied GPA BGCs and NovM from clorobiocin BGC [57], used as an outgroup (the tree is not drawn to scale). MEGA 11 was used for the analysis [58]. Evolution was inferred by using the JTT matrix-based model [59]. A discrete γ distribution was used to model evolutionary rate differences among sites (5 categories). Numbers at nodes indicate bootstrap-support values derived from 1000 replications. Five clades were delineated on this tree (**A**–**E**); clades correlated with substrate- and regiospecificity of GTFs. Veg36-OLZ52430-AIG79202 seemed to be a clade of few additional irrelevant GTFs coded nearby the corresponding BGCs. Notably, Tei1 (marked with “Glc*N*Ac-AA6 (!)” label) appeared in the clade (**A**) grouping GTFs attaching either d-glucose or Glc*N*Ac to aglycone AA4, while the enzyme was experimentally shown to attach Glc*N*Ac to aglycone AA6. “(?)” signs indicate presumed regiospecificity (lack of experimental evidence).

Teicoplanin and A40926 are chemically similar GPAs that likely emerged in the course of convergent evolution (Figure S5) [47,48,49]. Differences lie in the chlorination and methylation pattern; notably, A40926 also lacks a *N*-acetyl glucosamine (Glc*N*Ac) moiety attached to the aglycone of teicoplanin at the aa position 6 (AA6) (Figure S5). The glycosylation pattern might be important for binding VanS, in accordance with previous results reporting that VanS from *S. toyocaensis* was unable to recognize vancomycin, sensing only the non-glycosylated A47934 [33]. Hence, it could be speculated that the responsiveness of Tei3 to a GPA lacking a Glc*N*Ac residue might be an ancient property from the times when the ancestral teicoplanin BGC did not carry a gene for the attachment of the Glc*N*Ac moiety at aglycone AA6. To understand this better, the phylogeny of glycosyl transferases (GTFs) coming from experimentally studied GPA BGCs was reconstructed (Figure 6).

**Figure 7:**
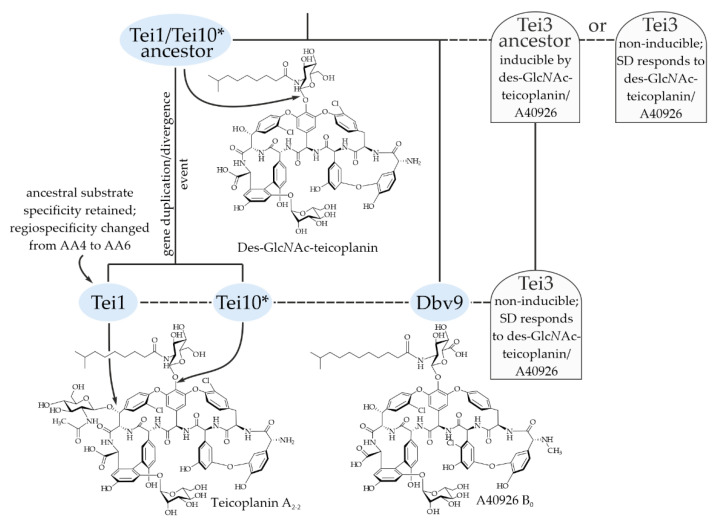
A scheme illustrating possible scenarios for the co-evolution of GTFs encoded in teicoplanin BGC and Tei3. Please refer to the main text for more detail.

Five clades (A–D) might be delineated on the obtained tree (Figure 6) and they seem to correspond to the regiospecificity of GTFs well. The regiospecificity of clades (A–C) GTFs could be predicted with high confidence as, for many members of these clades, experimental evidence exists. Thus, clade (A) comprehended GTFs attaching either d-glucose or Glc*N*Ac to AA4 of the GPA aglycone (see [50] for the review of such GTFs); clade (B) GTFs are responsible for the attachment of l-aminosugars to AA4 d-glucose [50]; clade (C) included GTFs from ristocetin BGCs likely attaching d-arabinose to AA4 d-mannosyl-d-glucose [51] (Figure 6). The substrate- and regiospecificity of GTFs from clades (D) and (E) were dubious: clade (D) GTFs probably attach l-rhamnose to the AA4 d-glucose, while clade (E) GTFs may attach l-aminosugars to aglycone AA6. To our surprise, Tei1—known to attach the Glc*N*Ac moiety at the teicoplanin aglycone AA6 [52]—was found deep in clade (A), being a sister branch to Tei10* (known to attach Glc*N*Ac at AA4). One other GPA—GP1416 from *Amycolatopsis* sp. WAC01416 [53]—is known to closely resemble the teicoplanin structure, bearing the Glc*N*Ac moiety at AA6. However, the GTF that is likely responsible for this did not belong to any of the clades and was located far from either Tei1 or Tei10* (Figure 6). The presence of the Glc*N*Ac moiety at AA6 of teicoplanin and GP1416 could, thus, be considered an example of the convergent evolution of GPAs. Considering all mentioned above, one could reasonably assume that Tei1 is a recent product of the Tei10* duplication/divergence event. In the course of evolution, it is likely that Tei1 changed its regiospecificity but retained the substrate specificity. A direct ancestor of teicoplanin BGC likely coded the biosynthesis of des-Glc*N*Ac-teicoplanin, which structurally resembles A40926 and was recognized by the Tei3 SD (see Figure 7 and the discussion below for a possible reconstruction of co-evolution of GTFs encoded in teicoplanin BGC and Tei3).

## 3. Discussion

*vanHAXRS* genes were experimentally shown to provide GPA resistance in (i) GPA-producing actinobacteria [34], (ii) actinobacterial GPA non-producers [31], (iii) GPA-resistant pathogens [28], and (iv) other soil bacteria [19,54]. In the majority of the known cases, the VanRS two-component regulatory system is utilized to sense extracellular GPAs [29] (or perhaps the GPA-lipid II complex [30]) and activate the expression of functional *van* genes. Pathogens and GPA-non-producing actinobacteria generally have inducible *van*-resistant phenotypes, because the constitutive expression of *van* genes comes at a cost of decreased fitness of the cells [55,56].

In contrast, the GPA resistance phenotype is commonly constitutive in GPA producers, as it was shown in *A. teichomyceticus* and *Am. balhimycina* [34]. In *Am. balhimycina*, *vanHAX* orthologs are the main contributors to GPA resistance [40,60]. However, *vanHAX* are not situated within the borders of balhimycin BGC (as commonly occurs in the other actinomycetes producing GPAs), and they are not co-localized with *vanRS* orthologs [40]. Hence, the expression of *vanHAX* is devoid of *vanRS*-mediated regulation and is constitutive. It should be noted that *Am. balhimycina* also possesses additional GPA-resistance mechanisms that are not *van*-mediated [61].

The most striking outcome of our work was that the SD of Tei3 did not respond to teicoplanin, but recognized A40926: the expression of the hybrid SHK carrying the Tei3 SD fused with the catalytic part from VanS_Sc_ changed the constitutive GPA-resistance phenotype of *S. coelicolor* J3200, making the strain teicoplanin- and vancomycin-sensitive, but A40926-resistant. This prompted us to focus on the chemical differences in the structure of A40926 and teicoplanin (the presence of a Glc*N*Ac residue in teicoplanin, which is absent in A40296) and on the possible evolution of the teicoplanin glycosylation pattern and of the whole *tei* BGC. Reconstruction of the phylogeny of GTFs coded within the known GPA BGCs allowed us to build possible scenarios explaining the obtained results (Figure 7).

If our reconstruction of the evolution of Tei1/Tei10* is correct, the ancestor of the teicoplanin BGC coded the biosynthesis of des-Glc*N*Ac-teicoplanin, which structurally resembles A40926 and was recognized by the Tei3 SD. Tei3 SHK initially responded to des-Glc*N*Ac-teicoplanin (modeled with A40926 in the experiment presented in this work) (Figure 7) and it was probably inducible. A further two alternative scenarios are possible. The first implies that the non-inducibility of Tei3 evolved as an adaptation to the appearance of Tei1 because *A. teichomyceticus* was required to rapidly gain constitutive GPA resistance as Tei3 was unable to sense teicoplanin. The second scenario implies that Tei3 in the des-Glc*N*Ac-teicoplanin producer became non-inducible at the first place, consequently giving the BGC room for further evolution and structural changes of the produced GPA. Considering that, in the first hypothesis, fewer mutations would probably be required to give Tei3 constitutive properties than to remodel its SD, the second scenario is more likely in our opinion. Hence, mutations leading to the non-inducibility of Tei3 might have served as a preadaptation, which allowed teicoplanin to emerge in its current form, carrying the Glc*N*Ac residue at AA6 (Figure 7).

To conclude, our results proved the non-inducibility of Tei3, showed that its SD is able to respond to A40926, but not to teicoplanin or vancomycin, and demonstrated a novel example of how BGCs, antibiotic structures, and antibiotic resistance genes might have co-evolved. Further investigations will include mutational analysis of Tei3 to elucidate which particular aa changes in its ATPaseD lead to non-inducible properties. Finally, as a byproduct, a *S. coelicolor*-based chromogenic assay for vancomycin detection was developed capable of sensing vancomycin at the ng range.

## 4. Materials and Methods

### 4.1. Plasmids, Bacterial Strains, and Cultivation Conditions

All plasmids and bacterial strains utilized or generated in the course of the current study are summarized in Table 1. For routine maintenance, *A. teichomyceticus* was cultivated on ISP3 agar [62] at 30 °C, and *S. coelicolor* strains were cultivated on SFM [45] agar at 30 °C. For genomic DNA isolation, *A. teichomyceticus* was cultivated in ISP2 liquid medium [47] in 50 mL baffled flasks on an orbital shaker at 200 rpm and 30 °C; *S. coelicolor* strains for genomic DNA isolation were cultivated under the same conditions in TSB medium [62]. Antibiotic susceptibility tests with *S. coelicolor* strains were performed on SMMS medium [45]. Apramycin sulfate (50 μg/mL), spectinomycin hydrochloride (50 μg/mL), and nalidixic acid (30 μg/mL) were used for the selection and maintenance of *S. coelicolor* recombinant strains. Vancomycin, teicoplanin, and A40926 were added to solid or liquid cultures at the desired concentrations reported in Section 2. *E. coli* DH5α was used as a general cloning host, while *E. coli* ET12567 pUZ8002^+^ was used as a donor for intergeneric matings. *E. coli* strains were cultivated at 37 °C in lysogeny broth or agar media supplemented with 100 μg/mL of apramycin sulfate, 50 μg/mL of kanamycin sulfate, and 25 μg/mL of chloramphenicol when appropriate. All antibiotics were purchased from Sigma-Aldrich (Merck Group, Darmstadt, Germany).

### 4.2. Generation of Recombinant Plasmids for Gene Expression and Promoter-Probe Vectors

In all cases described below, Q5 High-Fidelity DNA Polymerase (NEB, Ipswich, MA, USA) was used as a DNA polymerase of choice in PCRs according to the recommendations of the supplier. Restriction endonucleases and T4 DNA ligase from Thermo Fisher Scientific (Waltham, MA, USA) were always used for DNA digestion and ligation according to the supplier’s recommendations. Chromosome DNA, utilized as a PCR template, was isolated according to the Kirby procedure [45].

**pGP101, pGP111.** The coding sequence of the *tei3* gene was amplified from the chromosome DNA of *A. teichomyceticus* using the tei3_F/R primer pair (Table 2); the obtained 1133 bp amplicon was digested with *Eco*RI/*Eco*RV restriction endonucleases and cloned into the pSET152A vector digested with the same restriction endonucleases yielding pGP101. Further, pGP101 was digested with *Bam*HI/*Xho*I restriction endonuclease, and a 3247 bp DNA fragment carrying *aac(3)IVp-tei3* was purified; in parallel, the pRT801 plasmid was also digested with *Bam*HI/*Xho*I and the 3362 bp DNA fragment was purified. Both obtained fragments were then ligated, generating pGP111.

**pGP102, pKC1132-102, pKC1132-103, and pGP103.** The coding sequence of *vanSsc* (*SCO3589*) was amplified using the chromosome DNA of *S. coelicolor* M512 as a template with the SCO3859_F/R primer pair; the obtained 1130 bp amplicon was digested with *Eco*RI/*Eco*RV restriction endonucleases and cloned into pSET152A via the same sites, giving pGP102. pGP102 was digested with *Pvu*II and the 1753 bp fragment (carrying *aac(3)IVp-SCO3589*) was cloned into pKC1132 digested with the same restriction endonuclease, generating pKC1132-102. Then, a fragment of *SCO3589* coding for the SD was exchanged for the corresponding fragment of *tei3*. The fragment of the *tei3* sequence coding for the SD was amplified from pGP101 using the tei3_F and tei3_sdomain_PaeI primers pair; the obtained 285 bp amplicon was digested with *EcoR*V and *Pae*I restriction endonucleases. In parallel, pKC1132-102 was also digested with *EcoR*V and *Pae*I and a 4289 bp fragment was selected; this latter fragment was ligated with the *Pae*I-digested *tei3-SD* amplicon, yielding pKC1132-103. Finally, the hybrid ORF of *SCO3589* with the SD-coding fragment exchanged with *tei3-SD* was excised from pKC1132-103 using *Eco*RV and *Eco*RI restriction endonucleases, and cloned into pSET152A via the same recognition sites generating pGP103.

**pGP104, pGP105, pGP106, and pGP107.** These vectors carried truncated versions of *tei3* and *SCO3589* lacking parts of the sequence coding for (i) TMh1 (*tei3′*—pGP104; *SCO3589′*—pGP105) and (ii) whole SD (*tei3″*—pGP106; *SCO3589″*—pGP107). All these coding sequences were amplified using primers listed in Table 2, generating: *tei3′*—956 bp; *tei3″*—879 bp; *SCO3589′*—959 bp; *SCO3589″*—878 bp. Amplicons were digested with *Eco*RV/*Eco*RI and cloned into pSET152A via the same recognition sites.

**pGJp and pGHp.** DNA fragments including promoter regions of *S. coelicolor vanJ (SCO3592)* and *vanH (SCO3594)* genes were amplified from the chromosome DNA of *S. coelicolor* M512 using vanJp_KpnI_F/vanJp_SpeI_R and vanHp_KpnI_F/vanHp_SpeI_R primers pairs (Table 2). The obtained amplicons for *vanJp* (321 bp) and *vanHp* (539 bp) were digested with *Kpn*I/*Spe*I restriction endonucleases and ligated with pGUS digested by the same enzymes, yielding pGJp and pGHp promoter-probe vectors.

All generated plasmids were verified by restriction mapping and sequencing.

### 4.3. Conjugal Transfer of Recombinant Plasmids to S. coelicolor Strains

A standard protocol for the conjugal transfer of plasmids to *S. coelicolor* strains was utilized [45]. All necessary plasmids were individually transferred into the non-methylating *E. coli* ET12567 pUZ8002^+^ and the resulting derivatives were used as donor strains for intergeneric conjugation, while spores of *S. coelicolor* were used as acceptors. Circa 10^6^ spores were mixed with, ca., 10^9^ donor cells and plated on ISP3 agar supplemented with MgCl_2_ (10 mM); after 12 h of incubation at 30 °C, each plate was overlaid with 1 mL of sterile water with 1.25 mg of apramycin-sulfate and 750 μg of nalidixic acid. Transconjugants were selected as resistant to 50 μg/mL of apramycin sulfate or 50 μg/mL of spectinomycin hydrochloride. All recombinant strains were tested with PCR using chromosome DNA isolated according to the Kirby procedure [45]. *aac(3)IV* was amplified from chromosome DNA of transconjugants carrying pSET152A and pRT801 derivatives with the aac(3)IV_F/R primer pair. Transconjugants carrying promoter-probe vectors were tested by amplifying the 1000 bp internal region of *gusA* with the gusA_ver_F/R primer pair (Table 2).

### 4.4. Qualitative and Quantitative Glucuronidase Assays

Qualitatively, the β-glucuronidase (GusA) activity in *S. coelicolor* was assessed by adding 25 mg/mL of 5-bromo-4-chloro-3-indolyl-β-d-glucuronide (X-Gluc, Thermo Fisher Scientific, Waltham, MA, USA) to SMMS agar. The chromogenic conversion of X-Gluc into the green-colored 5,5′-dibromo-4,4′-dichloro-indigo was then monitored. Quantitative measurement was performed as follows. A number of 10^7^ spores of *S. coelicolor* pGJp^+^ and pGHp^+^ were inoculated into a baffled 300 mL Erlenmeyer flask containing 50 mL of TSB and incubated without and with vancomycin as an inducer. Mycelium obtained in this way was used to prepare cell-free lysates as reported previously [63]. Glucuronidase activity was measured in cell-free lysates as described previously [44,63] utilizing a spectrophotometric assay to detect the conversion of the colorless *p*-nitrophenyl-β-d-glucuronide (Thermo Fisher Scientific, Waltham, MA, USA) into the colored *p*-nitrophenol at 415 nm using a Unicam UV 500 UV-Visible Spectrometer (Thermo, Waltham, MA, USA). Glucuronidase activity was normalized to the weight of dry biomass, and one unit of activity was considered as the amount of enzyme able to convert 1 μM of the substrate in 1 min.

### 4.5. Tools for In Silico Analysis

Clustal Omega (EMBL-EBI) [41] was used for pairwise alignment of aa and nucleic acid sequences. CD-Search was used to identify conserved domain regions [42]. Geneious 4.8.5 was utilized for routine analysis of aa and nucleic acid sequences [64]. MEGA11 (v.11.0.13) was used to perform phylogenetic reconstruction [58].

## Figures and Tables

**Table 1:** Plasmids and bacterial strains used or generated in this study.

Name	Characteristic	Reference
Plasmids:		
pSET152A	φC31-based integrative plasmid, pSET152 derivative carrying *aac(3)IVp* from pIJ773, Am^r^	[63]
pKC1132	suicide vector, Am^r^	[45]
pRT801	φBT1-based integrative plasmid, Am^r^	[46]
pGUS	φC31-based integrative plasmid, pSET152 derivative containing promoterless GusA, Am^r^, Sp^r^	[44]
pGP101	pSET152A derivative carrying *tei3*	this work
pGP111	pRT801 derivative carrying *tei3*	this work
pGP102	pSET152A derivative carrying SCO3589 (*vanS_Sc_)*	this work
pKC1132-102	pKC1132 derivative carrying SCO3589 (*vanS_Sc_*)	this work
pKC1132-103	pKC1132 derivative carrying hybrid SHK gene with *tei3*-SD and *vanS_Sc_*-ATPaseD	this work
pGP103	pSET152A derivative carrying hybrid SHK gene with *tei3*-SD and *vanS_Sc_*-ATPaseD	this work
pGP104	pSET152A derivative carrying *tei3*′	this work
pGP105	pSET152A derivative carrying SCO3589′	this work
pGP106	pSET152A derivative carrying *tei3*″	this work
pGP107	pSET152A derivative carrying SCO3589″	this work
pGJp	pGUS derivative carrying *vanJp*	this work
pHJp	pGUS derivative carrying *vanHp*	this work
Strains:		
*A. teichomyceticus* ATCC 31121	wild type, teicoplanin producer	ATCC
*S. coelicolor* M512	A3(2) derivative, Δ*redD* Δ*actII-ORF4* SCP1^−^ SCP2^−^	[38]
*S. coelicolor* J3200	A3(2) derivative, Δ*SCO3589* (*vanS_Sc_*)	[31]
*S. coelicolor* M101	M512 derivative carrying pGP101	this work
*S. coelicolor* M111	M512 derivative carrying pGP111	this work
*S. coelicolor* pGJp^+^	M512 derivative carrying pGJp	this work
*S. coelicolor* pGHp^+^	M512 derivative carrying pGHp	this work
*S. coelicolor* M1J	M512 derivative carrying pGJp and pGP111	this work
*S. coelicolor* M103	M512 derivative carrying pGP103	this work
*S. coelicolor* M104	M512 derivative carrying pGP104	this work
*S. coelicolor* M105	M512 derivative carrying pGP105	this work
*S. coelicolor* M106	M512 derivative carrying pGP106	this work
*S. coelicolor* M107	M512 derivative carrying pGP107	this work
*S. coelicolor* J103	J3200 derivative carrying pGP103	this work
*S. coelicolor* J104	J3200 derivative carrying pGP104	this work
*S. coelicolor* J105	J3200 derivative carrying pGP105	this work
*S. coelicolor* J106	J3200 derivative carrying pGP106	this work
*S. coelicolor* J107	J3200 derivative carrying pGP107	this work

**Table 2:** Oligonucleotide primers used in this study.

Name	Sequence (5′-3′) *	Purpose
tei3_sdomain_PaeI	CGAGCATGCGACCGGCCAG	Reverse primer for cloning the *tei3* region coding for the sensor domain
tei3_F	TTTGATATCGGAGGGAGACCGTGGACCGAGCCC	Cloning of the *tei3* and its truncated versions
tei3’_F	TTTGATATCGGAGGGAGACCGTGTTCGCCCCGGCGACGG	-//-
tei3”_F	TTTGATATCGGAGGAGACCGTGGGTCGGATGCTCGCTCCTCT	-//-
tei3_R	TTTGAATTCGCGGTGGGCGGTTCAGTTT	-//-
SCO3589_F	TTTGATATCGGAGGGCGACGGTGGATAGGCGCC	Cloning of the *SCO3589 (vanS_Sc_)* and its truncated versions
SCO3589’_F	TTTGATATCGGAGGGAGACCGTGCTTCGCAGTTTCGCCC	-//-
SCO3589’’_F	TTTGATATCGGAGGGCGACGGTGGGACGCATGCTCGCCCCCCT	-//-
SCO3589_R	TTTGAATTCTGGCGCTCACCTGCCGGTG	-//-
vanHp_KpnI_F	TTTGGTACCTACGTCCACACCGCCGAGC	Cloning of *vanH_Sc_* promoter region into pGUS
vanHp_SpeI_R	TTTACTAGTGCCGTCCCGTATGCGCTTT	-//-
vanJp_KpnI_F	TTTGGTACCACACTCAGCAGCTCCAACG	Cloning of *vanJ_Sc_* promoter region into pGUS
vanJp_SpeI_R	TTTACTAGTCTGGCGCCGGTGCGGCCGA	-//-
aac(3)IV_F	ATCGACTGATGTCATCAGCG	Diagnostic primers for the amplification of *aac(3)IV* gene
aac(3)IV_R	CGAGCTGAAGAAAGACAAT	-//-
gusA_ver_F	GGCGGCTACACGCCCTTCGA	Diagnostic primers for the amplification of 1000 bp internal region of *gusA*
gusA_ver_R	TGATGGGCCGGGTGGGGTC	-//-

* artificial ribosome binding sites are highlighted in red and artificial start codons are underlined.

## Data Availability

All the data are available from the corresponding author upon reasonable request.

## Supplementary Materials

The following supporting information can be downloaded at: https://www.mdpi.com/article/10.3390/ijms232415713/s1.
