# Supplementary material for: Heterologous Expression Reveals Ancient Properties of Tei3—A VanS Ortholog from the Teicoplanin Producer Actinoplanes teichomyceticus
Source: Int J Mol Sci. 2022 Dec 11;23(24):15713. doi: 10.3390/ijms232415713 (PMC9779433; doi:10.3390/ijms232415713)
Supplement: Supplementary file 1 [file ijms-23-15713-s001.zip › supplementary materials.pdf]

Electronic supplementary materials

Heterologous expression reveals ancient properties of Tei3 – a VanS orthologue from the teicoplanin producer *Actinoplanes teichomyceticus*

Oleksandr Yushchuk [1,2], Kseniia Zhukrovska [2], Bohdan Ostash [2], Victor Fedorenko [2], and Flavia Marinelli [1*]

[1] Department of Biotechnology and Life Sciences, University of Insubria, 21100 Varese, Italy

[2] Department of Genetics and Biotechnology, Ivan Franko National University of Lviv, 79005 Lviv, Ukraine

*Correspondence: flavia.marinelli@uninsubria.it; Tel.: +39-0332-42-1546

Supplementary figures

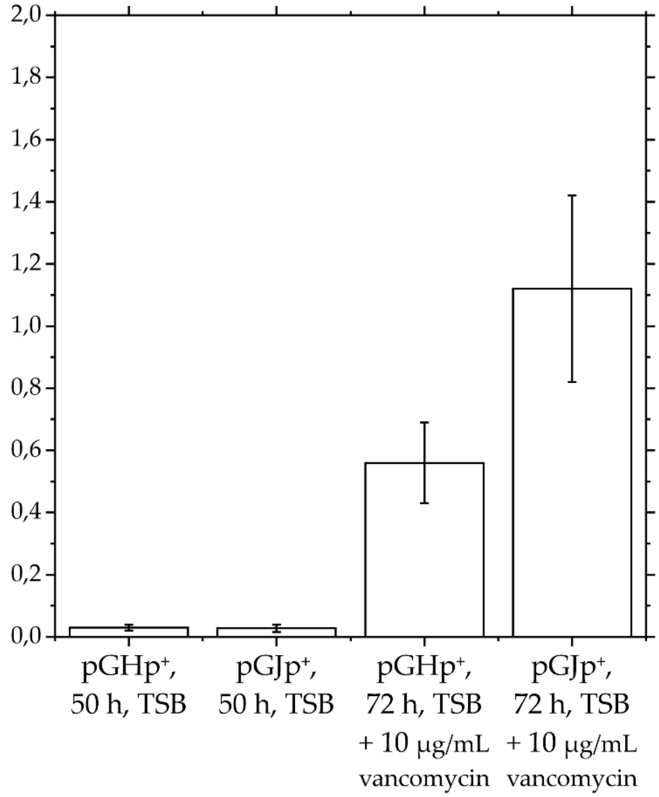

Figure S1. *S. coelicolor* M512 derivatives carrying promoter-probe vectors pGHp and pGJp are able to convert *p*-nitrophenyl-β-D-glucuronide to *p*-nitrophenol in response to the addition of vancomycin. The two strains were cultivated in TSB medium for 50 h and samples of the mycelium were collected at this time-point. After that, vancomycin was added to the broth up to 10 μg/mL and the strains were left for up to 72 h of cultivation. Glucuronidase activities of cell-free lysates from mycelium obtained before and after vancomycin induction were compared in a quantitative glucuronidase assay.Quantitative measurement of GusA activity (U/gram of dry biomass) in *S. coelicolor* pGHp+ and pGJp+ was measured as described in Materials and Methods section.

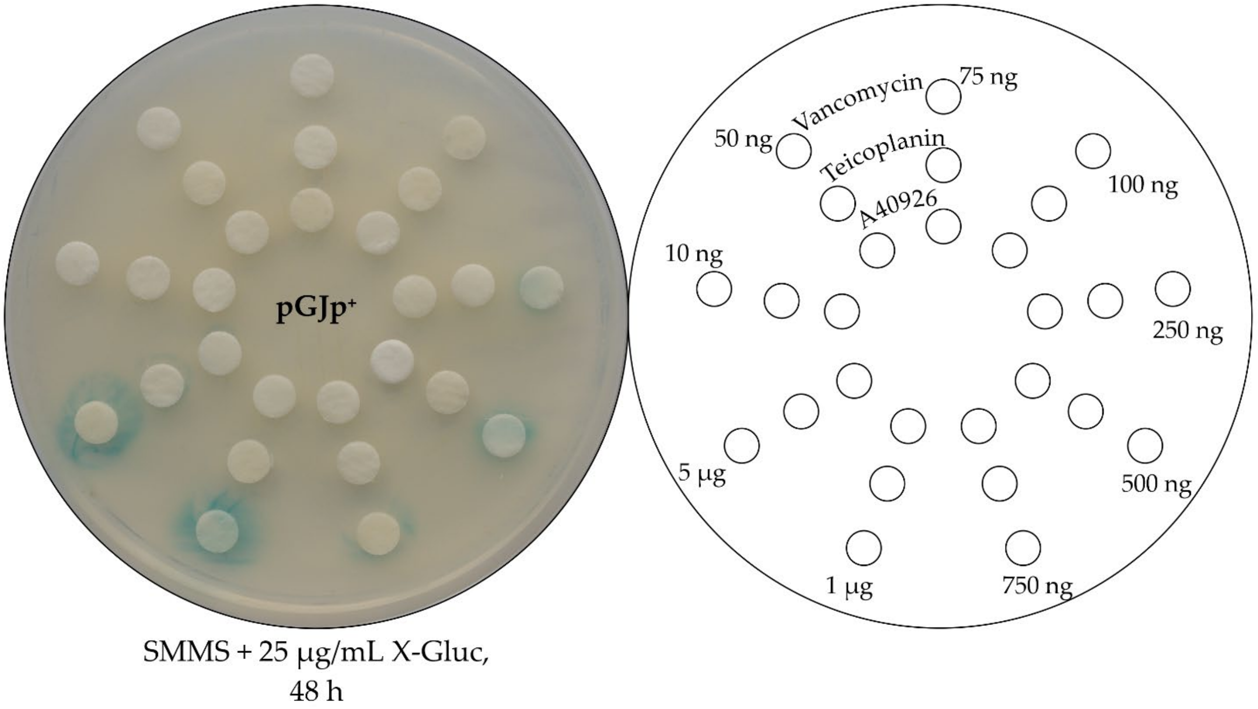

Figure S2. Qualitative chromogenic assay showing *S. coelicolor* pGJp+ able to convert 5-bromo-4-chloro-3-indolyl-β-D-glucuronide (X-Gluc) to 5,5′-dibromo-4,4′-dichloro-indigo (green colour) in response to GPAs. *S. coelicolor* M512 derivative carrying promoter-probe vectors pGJp was grown as a confluent lawn on SMMS agar containing 25 μg/mL X-Gluc. After 48 h growth, Whatman discs soaked with different concentrations of vancomycin, teicoplanin, and A40926 were deposited on the surfaces of the solid culture. The green halos around the Whatman discs indicate of the glucuronidase activity due to the induction of the *vanJp*. Only 250 ng of vancomycin were able to induce the chromogenic conversion of X-Gluc.

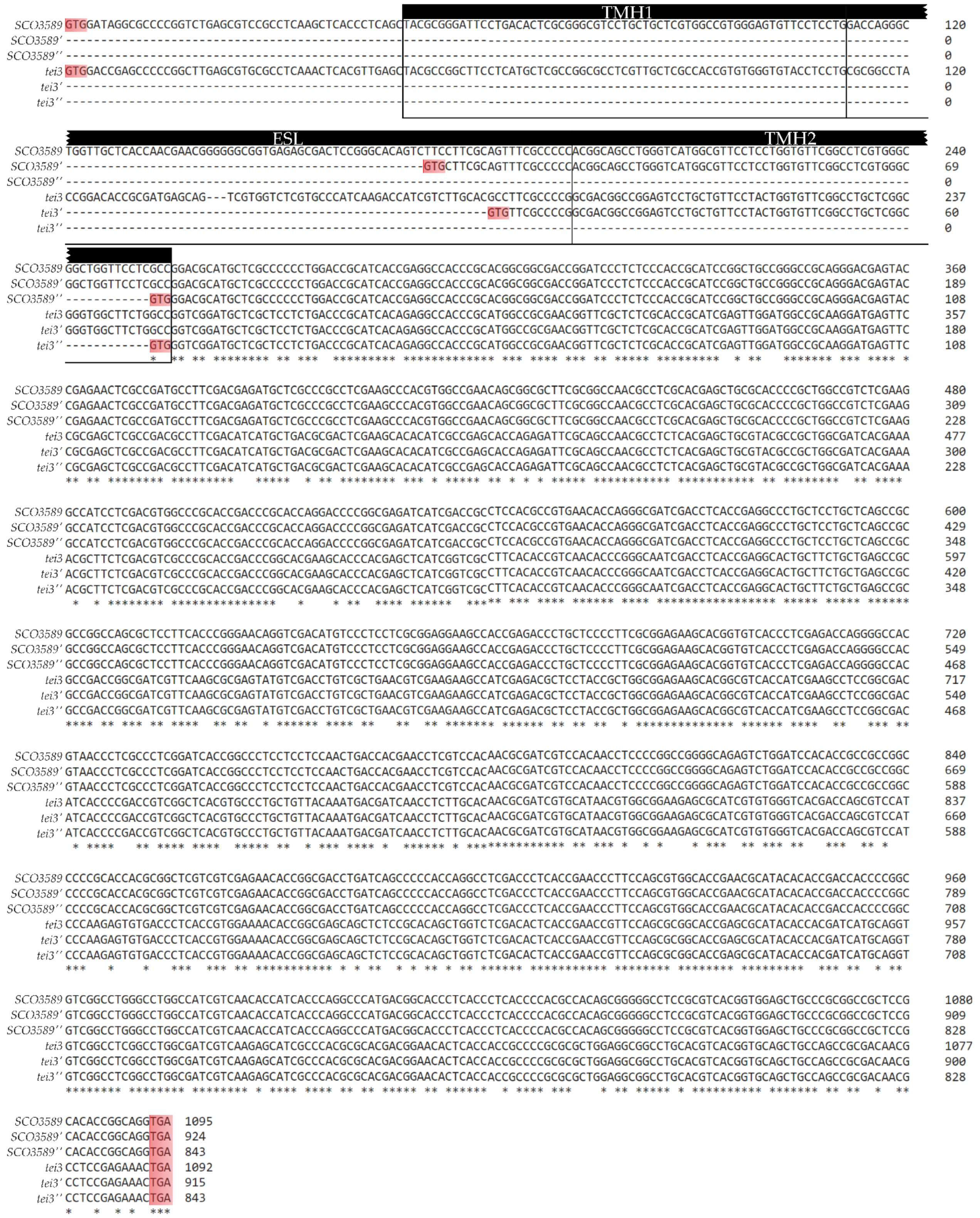

Figure S3. Clustal Omega multiple sequence alignment of the truncated versions of *tei3* and *SCO3589* used in this study; start and stop codons are highlighted in red, transmembrane helices (TMH1 and TMH2) as well as extracytoplasmic sensory loop (ESL) are shown.

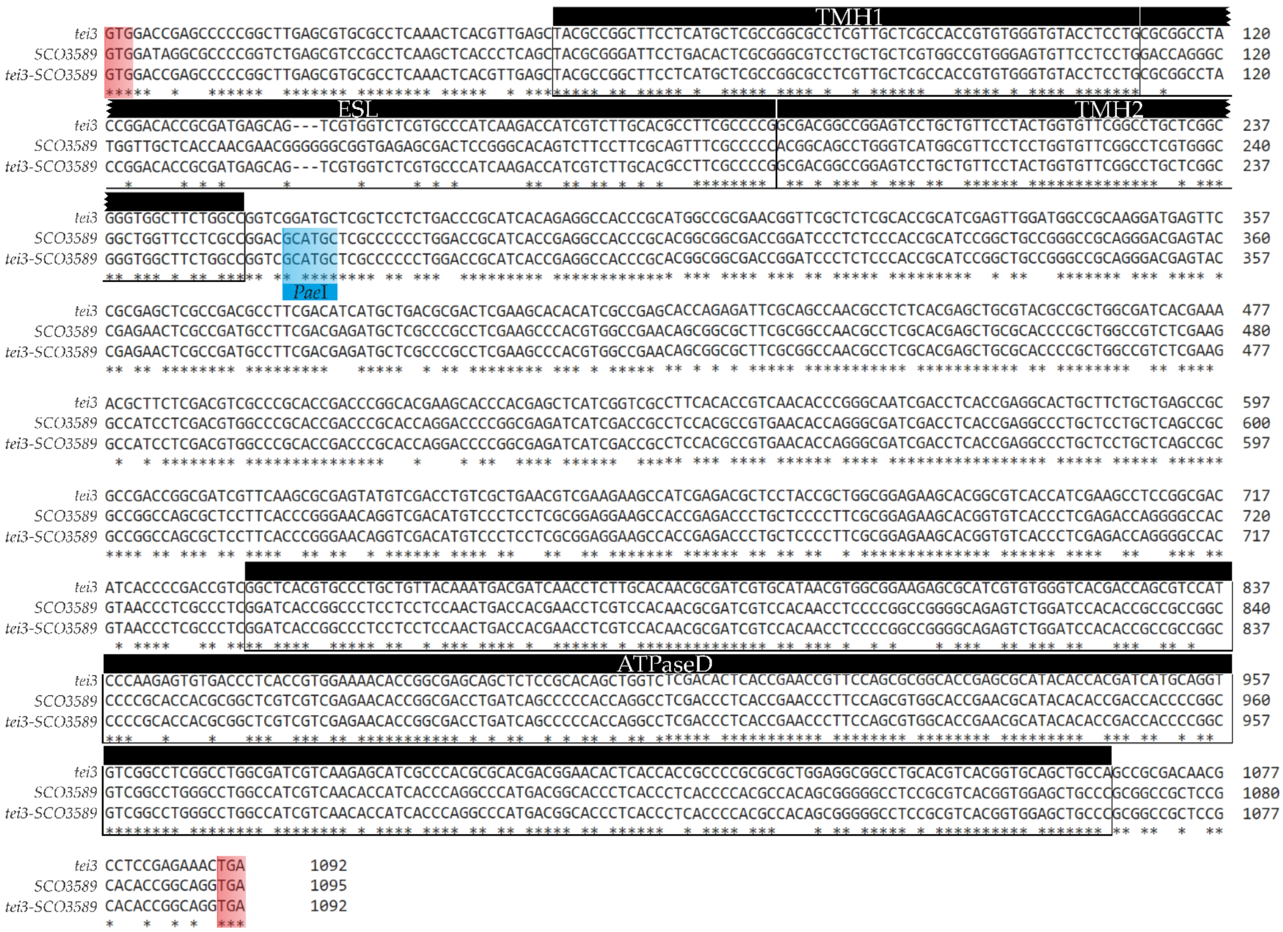

Figure S4. Clustal Omega multiple sequence alignment of the *tei3, SCO3589* and their hybrid. Start and stop codons are highlighted in red; *Pae*I recognition site is highlighted in blue; transmembrane helices (TMH1 and TMH2) as well as extracytoplasmic sensory loop (ESL) and ATPase domain (ATPaseD) are shown.

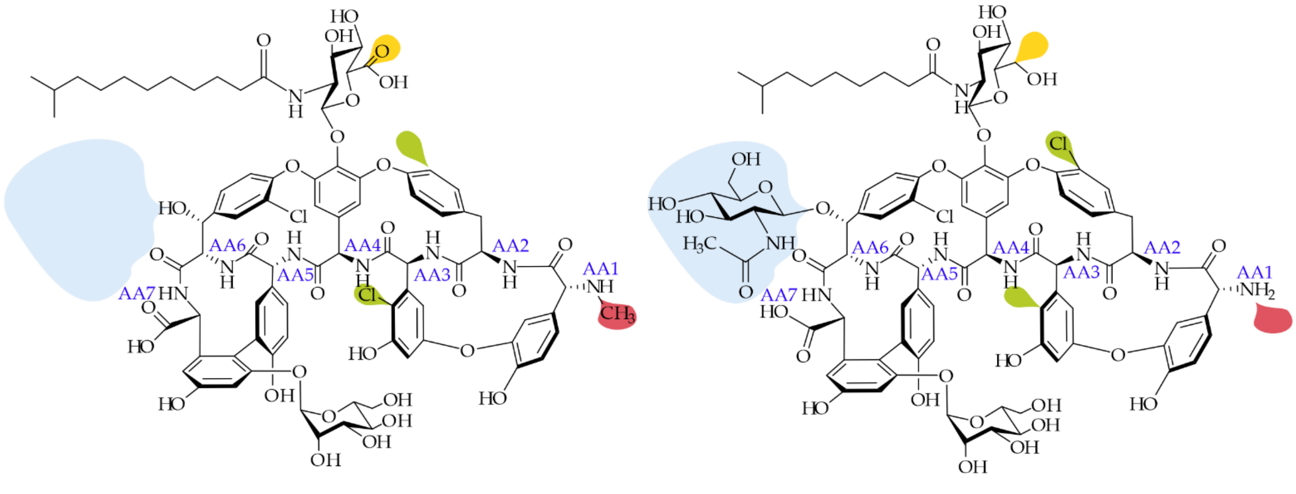

A40926 B_0 Teicoplanin $A_{2\text{-}2}$

Figure S5. Chemical structures of A40926 B_0 and teicoplanin $A_{2\text{-}2}$ (main congeners produced in the corresponding natural complexes). The main differences in the structure of these two GPAs lie in chlorination pattern (highlighted in green), methylation (pink), carboxylation of AA4 *N*-acylglucosamine (yellow), and the presence of Glc*N*Ac moiety at AA6 (blue).
